# Design, Synthesis and Discovery of *N,N’*‐Carbazoyl‐aryl‐urea Inhibitors of Zika NS5 Methyltransferase and Virus Replication

**DOI:** 10.1002/cmdc.201900533

**Published:** 2020-01-07

**Authors:** Sharon Spizzichino, Giulio Mattedi, Kate Lauder, Coralie Valle, Wahiba Aouadi, Bruno Canard, Etienne Decroly, Suzanne J. F. Kaptein, Johan Neyts, Carl Graham, Zakary Sule, David J. Barlow, Romano Silvestri, Daniele Castagnolo

**Affiliations:** ^1^ School of Cancer and Pharmaceutical Sciences King's College London London SE1 9NH UK; ^2^ Department of Drug Chemistry and Technologies Sapienza University of Rome Laboratory Affiliated to Instituto Pasteur Italia – Fondazione Cenci Bolognetti Piazzale Aldo Moro 5 00185 Roma Italy; ^3^ AFMB, CNRS Aix-Marseille University UMR 7257, Case 925 163 Avenue de Luminy 13288 Marseille Cedex 09 France; ^4^ Department of Microbiology, Immunology and Transplantation Rega Institute for Medical Research Laboratory of Virology and Chemotherapy KU Leuven Minderbroedersstraat 10 3000 Leuven Belgium

**Keywords:** Zika, flavivirus, methyltransferase, antiviral agents, urea

## Abstract

The recent outbreaks of Zika virus (ZIKV) infection worldwide make the discovery of novel antivirals against flaviviruses a research priority. This work describes the identification of novel inhibitors of ZIKV through a structure‐based virtual screening approach using the ZIKV NS5‐MTase. A novel series of molecules with a carbazoyl‐aryl‐urea structure has been discovered and a library of analogues has been synthesized. The new compounds inhibit ZIKV MTase with IC_50_ between 23–48 μM. In addition, carbazoyl‐aryl‐ureas also proved to inhibit ZIKV replication activity at micromolar concentration.

## Graphical abstract

**Novel antivirals targeting ZIKA virus**: The recent outbreaks of Zika virus (ZIKV) worldwide make the discovery of novel antivirals a priority. Using a structure‐based virtual screening approach we were able to identify and develop a novel series of carbazoyl‐urea derivatives that are able to inhibit the ZIKV NS5‐MTase as well as ZIKV replication at micromolar concentration.

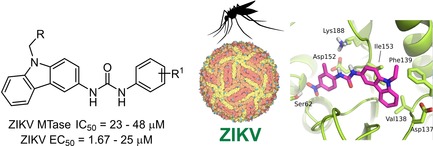

Zika virus (ZIKV)^1^, ^2^ is a single positive‐strand RNA mosquito‐borne pathogen belonging to the family of *Flaviviridae*, genus *Flavivirus* and it causes mild‐severe diseases in humans and animals. ZIKV is closely related to Dengue virus (DENV), and just like DENV, is mainly transmitted to humans by bites of an infected *Aedes s*pecies mosquitoes (*Ae. aegypti* and *Ae. albopictus)*.^3^ However, ZIKV can also be transmitted between humans through contact with blood or other body fluids, sexual transmission^2^, ^4^ and by mother‐to‐fetus transmission during the pregnancy.^6^ While global epidemics of DENV have spread over the past few decades causing more than 20,000 demises per year, ZIKV infections have emerged as a major public health concern only in the last few years. Before 2007, only sporadic human disease cases were reported in Africa and Asia. However, following the recent outbreaks in the Americas in 2015, ZIKV was declared by the World Health Organization (WHO) as a Public Health Emergency of International Concern (PHEIC) and the first case of sexually‐transmitted ZIKV was reported in USA in 2008. While ZIKV infections generally cause a mild fever, headache, malaise, skin rashes and joint pain, they have been associated in several cases with severe neurological and fetal complications leading to microcephaly in new‐borns^4^ and neurological diseases, such as the Guillain–Barré syndrome (GBS), in adults.^5^ The recent *Flavivirus* outbreaks, as well as the increasing number of cases of ZIKV infections worldwide, have raised the attention of pharmaceutical industries and healthcare providers toward the identification and development of efficient treatments against these diseases. Although a vaccine against DENV has recently been commercialized (DENGVAXIA^®^),^6^ there is as yet no vaccine against ZIKV available. In addition, there are no drugs available to treat or prevent ZIKV infections, especially in the event of an outbreak. A limited number of early‐phase discovery studies have identified few inhibitors of DENV and ZIKV replication,^7^, ^8^, ^9^, ^10^, ^11^, ^12^, ^13^, ^14^, ^15^ however there are no clinically approved drugs yet available to target flaviviruses directly nor any that may serve as vaccine adjuvants. The development of new antivirals thus represents a research priority.

Within this context, viral proteins represent appealing targets for the development of novel antiviral therapies. Examples of current flavivirus inhibitors, such as **1**–**5** (Figure 1), have been designed to target the DENV viral proteins NS3 (protease domain,^16^ helicase domain,^17^ and full‐length NS3), NS5 (N‐terminal Methyltransferase domain (MTase)^18^, ^19^ and C‐terminal RNA‐dependent RNA polymerase (RdRp).^20^ The NS3 and NS5 proteins of DENV and ZIKV show a high degree of homology, and their crystal structures have recently been determined.^21^, ^22^, ^23^ While DENV NS5‐polymerase has been investigated by a number of research groups as a potential target for development of new antivirals, there has been very little work carried out on DENV and ZIKV NS5‐MTase.^18^, ^23^, ^24^ The NS5‐MTase is responsible for maturation of the viral RNA cap and catalyzes the methylation of the N7 position of a guanine and the 2′‐OH of the first ribonucleotide to yield^7Me^GpppA_2′*O*Me_‐RNA.^25^ These methylations of the RNA cap structure play a key role during virus replication and are critical to virus survival in infected animals.^26^ Indeed, biochemical studies and reverse genetic analysis have shown that N7‐MTase activity is essential for mRNA translation into viral protein, and so viruses devoid of N7‐MTase show a strongly reduced replication phenotype.^18^ By contrast, 2′‐*O*‐MTase defective viruses can replicate moderately well in infected cells, but are highly attenuated in mice or rhesus monkey and induce a strong antiviral response.^27^ Thus, the inhibition of both N7‐ and 2′‐*O*‐MTase activities should restrain viral replication, making the NS5‐MTase a promising target for the development of new anti‐ZIKV, and potentially anti‐DENV, antivirals.^24^

**Figure 1:**
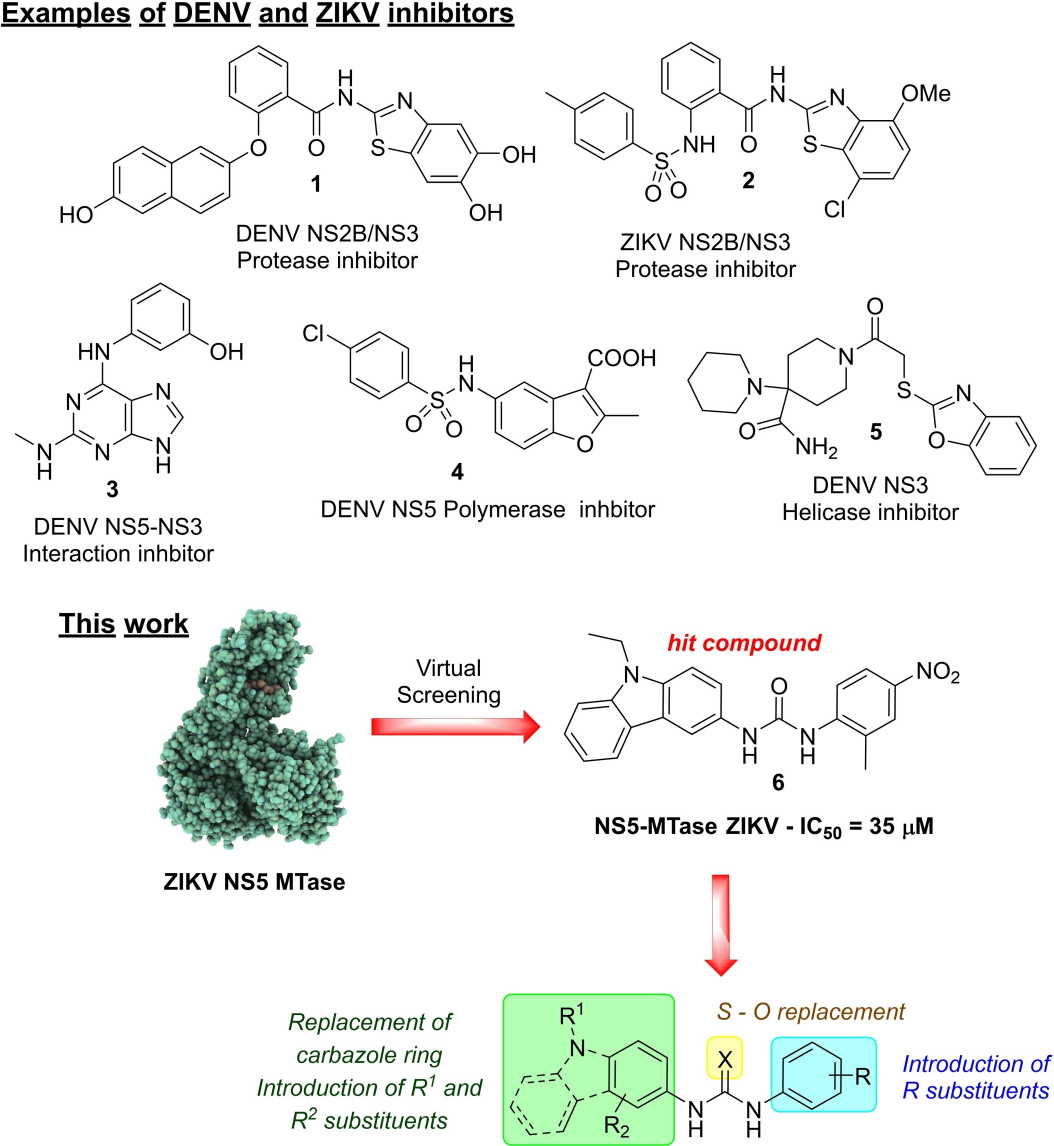
Examples of small molecules active against DENV and/or ZIKV, and overview of this work.

Herein, a structure‐based virtual screening on a set of chemical libraries using the ZIKV NS5‐MTase was performed, with the aim to identify structurally novel flaviviruses inhibitors.

The structure of the ZIKV NS5 MTase was built by homology modelling using SWISS‐MODEL^28^, ^29^ and the compounds in the NCI Diversity Set V database docked in the S‐adenosyl methionine (SAM) binding site in order to identify molecules that might disrupt the activity of the enzyme. A set of 40 best ranking molecules were initially identified, among which the urea **6** emerged as the best candidate due to its favorable interactions with the SAM binding site. The urea **6** was thus evaluated in enzymatic inhibition assays against ZIKV NS5‐MTase, showing an IC_50_ of 35 μM (Table 1). A similar IC_50_ of 38 μM was observed when **6** was assayed against DENV NS5‐MTase. Due to the novelty of the structure when compared to known flavivirus inhibitors, the urea **6** was thus selected as a hit compound for further studies. Figure 2b shows the most favorable binding pose of the hit compound **6** in the ZIKV NS5‐MTase binding pocket. The urea spacer connects the carbazole with a 2‐methyl‐4‐nitro‐phenyl group, which occupies the region that interacts with the methionine backbone of SAM (Figure 2a). The nitro group of **6** interacts with the side chain of S62 in a similar way to the SAM carboxyl group, possibly stabilizing the interaction of the compound with the protein. The high sequence and structural identity between ZIKV and DENV NS5‐MTases led to the identical binding of compound **6** in both proteins (Figure 2c).

**Table 1:** IC_50_ values of compounds **6**, **9**, **11** and **21** against host and flavivirus MTases.

Compd	hRNMT	ZIKV NS5‐MTase	DENV NS5‐MTase
	IC_50_ [μM]		
6	13.9±0.7	35±5	38±6.7
9a	≈122	46±1.1	≈133
9b	≈253	≈144	≈267
9c	27.4±1.9	NA^[b]^	≈62
9d	5.3±0.4	≈397	60±32
9f	6.8±0.5	NA^[b]^	NA^[b]^
11b	≈112	70±1.3	≈236
21b	Nd^[a]^	114±1.3	NA^[b]^
21c	nd^[a]^	23±1.2	NA^[b]^
21e	nd^[a]^	48±1.3	NA^[b]^
21f	nd^[a]^	26±1.2	NA^[b]^
Sinefungin	nd^[a]^	1.18±0.05	0.63±0.04

[a] Activity not determined. [b] No activity observed above 50 μM.

**Figure 2:**
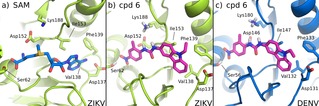
a) SAM bound ZIKV NS5 MTase in PDB 5M5B.^23^ b) Docking of compound **6** in ZIKV NS5 MTase. The carbazole pocket is accommodated in the hydrophobic environment that binds the adenine portion of SAM. The *p*‐nitro group interacts with Ser62, mimicking the carboxy moiety of SAM. The carbamide spacer allows the two domains to be spaced appropriately for favorably interacting with the key residues of the binding pocket. c) Docking of compound **6** in DENV NS5‐MTase (PDB 5E9Q).^24^

Next, a library of analogues of compound **6** was designed and synthesized to explore the chemical space around the urea scaffold (Scheme 1). The carbazole derivatives **9 a**–**f** were first synthesized through reaction of carbazole **7** with the appropriate isocyanate **8** in toluene at 60 °C with the aim to investigate the effect of different substituents (electron‐donating and electron‐withdrawing) on the phenyl ring bound to the urea moiety (Scheme 1a). Similarly, thioureas **11 a**–**b** were synthesized from **10 a**–**b** to investigate the role of the isosteric sulfur in place of the urea oxygen atom and to evaluate the influence of an extra methylene group in **11 b** on MTase inhibition (Scheme 1b).

**Scheme 1:**
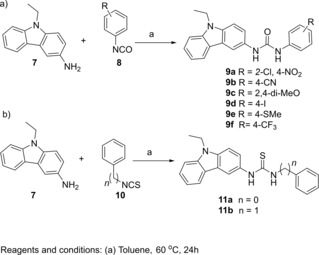
Synthesis of urea derivatives **9 a**–**f** and **11 a**,**b**.

The role of the carbazole ring on the antiviral activity was also explored through its replacement with various phenyl moieties (derivatives **12 a**–**e**) and indole ring (derivatives **14 a**–**d**). Ureas **12 a**–**e** and **14 a**–**d** were obtained from appropriate isocyanates **8** and **10** in toluene according to Scheme 2.

**Scheme 2:**
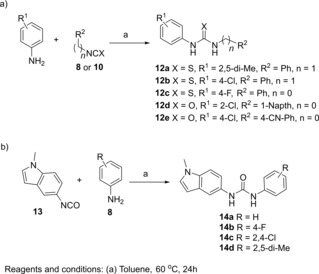
Synthesis of urea derivatives **12 a**–**e** and **14 a**–**d**.

In order to evaluate the role of the nitro group on antiviral activity, the amino derivatives **15** and **16** were also synthesized; compounds **6** was treated with Fe^(0)^ in conc. HCl leading to **15** in high yields, while carbazole **7** was reacted under microwave irradiation with Cbz,Boc‐lysine to give, after deprotection in HCl/AcOEt, the pure **16** (Scheme 3a). Finally, we also tried to modify the *N*‐ethyl substituent of **6** in order to increase the selectivity of our inhibitors targeting the MTase SAM binding site. For this purpose, we compared the structure of the human N7 MTase (hRNMT) involved in the capping of cellular mRNA, with that of both viral MTases. The X‐ray structure revealed structural similarities between the viral and the human MTases (NS5 and hRNMT). However, both ZIKV and DENV NS5‐MTase possess a hydrophobic pocket located near the exocyclic amide of SAM,^18^ which is absent in the hRNMT. In the ZIKV MTase, the region is defined by the amino acids F139, I153, G164, E155, R166, and V170, compared to residues F133, I147, G148, E149, R160, and V164 in the DENV MTase. We anticipated that the absence of this hydrophobic pocket in hRNMT could help to increase compound selectivity for the viral MTases and, hence, limit the interference of inhibitors with host MTases. In the docking snapshot, the ethyl group of compounds **6** is located in proximity of this pocket, clearly showing that its replacement with a benzyl or a phenylethyl moiety could lead to derivatives able to selectively recognize and inhibit only the viral enzymes over the host enzymes (Figure 3).

**Scheme 3:**
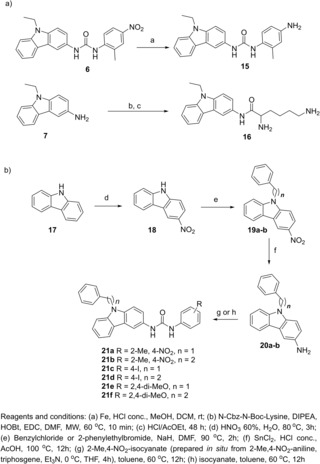
Synthesis of urea derivatives **15**, **16** and **21 a**–**f**.

**Figure 3:**
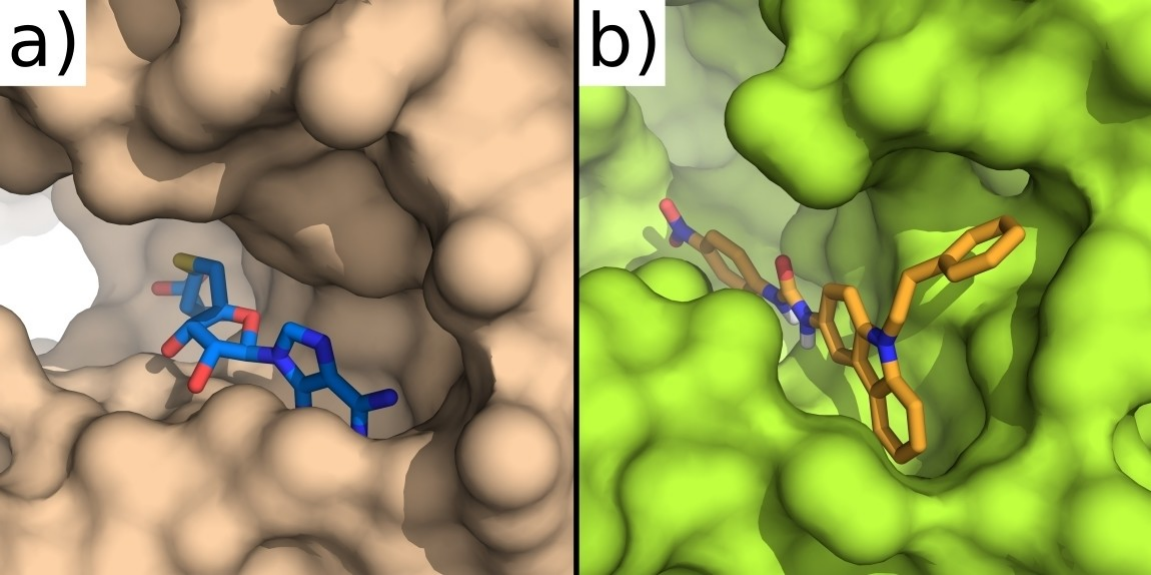
a) SAH bound to hRNTM (PDB 5E8J).^30^ b) Docking of compound **21 b** in ZIKV NS5 MTase. The ethyl spacer on the carbazole nitrogen allows the phenyl ring to interact effectively with the hydrophobic pocket near the SAM binding site. The absence of the region in hRNMT is a potential determinant of target selectivity.

Thus, the synthesis of derivatives **21 a**–**f** bearing a phenyl‐alkyl substituent on the carbazole nitrogen was planned. Carbazole **17** was treated with HNO_3_ and converted into the nitro derivative **18**, which was in turn alkylated on the aromatic nitrogen with appropriate benzyl or alkyl halide in the presence of NaH to give compounds **19 a**–**b**. Reduction of the nitro group of **19** with SnCl_2_ led to amino derivatives **20 a**–**b**. The latter were then treated with 4‐I‐phenyl‐ or 2,4‐(MeO)_2_‐phenyl‐isocyanate in toluene at 60 °C affording ureas **21 c**–**f** in high yields. A different strategy was adopted to synthesize the derivatives **21 a**–**b**, due to the commercial lack of the appropriate isocyanate reagent. The 2‐methyl‐4‐nitro‐aniline was treated with triphosgene at 0 °C in the presence of Et_3_N leading to the formation of the corresponding isocyanate, which was reacted *in situ* with carbazole **7** to give the desired ureas **21 a**–**b** in good yields (Scheme 3).

The library of compounds **9**, **11**, **12**, **14**, **15**, **16** and **21** was then assessed on different purified recombinant MTases. All compounds were initially screened at 50 μM against ZIKV NS5‐MTase and hRNMT as well as against DENV NS5‐MTase due to its similarity with ZIKV protein. The SAM mimetic sinefungin was included as control.

The MTases were incubated with radiolabeled [^3^H]‐SAM together with the GpppAC_4_ RNA substrate and the different compounds at 50 μM. The reaction was stopped after 30 mins at 30 °C. The sample products were filtered on a DEAE membrane to remove non‐incorporated [^3^H]‐SAM, and radioactivity transferred on the RNA substrate was counted. Compounds **6**, **9 a**, **9 d**, **21 b**, **21 c**, **21 e** and **21 f** showed inhibition of ZIKV NS5‐MTase higher than ∼30 %. The compound **21 e** and **21 f** showed the more potent reduction of ZIKV NS5‐MTase activity of ∼70 %.

Conversely the compounds **15** and **16** and those of series **11** and **14** barely inhibited the ZIKV MTase. Interestingly compound **6** and those of the series **9** showed a similar inhibition profile on DENV NS5‐MTase, but the compounds of series **21** barely inhibit the DENV MTase suggesting some specificity of this family. A dose‐response assay was then performed for the most promising compounds and the IC_50_ values deduced from titration curves after curve fitting are shown in Table 1. The results indicate that the replacement of the methyl group of **6** with a chlorine atom in **9 a** did not affect the inhibitory activity of the compound against ZIKV MTase, whilst it proved to be detrimental for inhibiting the DENV MTase. Similarly, the replacement of the electron withdrawing nitro group of **6** with other substituents (i. e. the electron donating iodine or methoxy in **9 c**–**d**, or electron withdrawing −CN or −CF_3_ in **9 b** and **9 f**) negatively affected the compound's inhibitory activity mainly against the ZIKV MTase. The different activity of compounds **6** and **9** can be explained by taking in consideration the crucial H‐bond interaction between nitro groups of **6** with the hydroxyl group of S62, which is not possible for derivatives **9 b**–**f**. The low activity of compounds **11 b** can be attributed to the lack of appropriate substituents on the phenyl ring able to interact with S62. On the other hand, the carbazole ring proved to be crucial for the antiviral activity since its replacement with other rings in **12** and **14** led to inactive compounds. Derivatives **21 c**, **21 e** and **21 f** showed good activity against ZIKV MTase at concentrations similar to **6** (IC_50_=23–48 μM). It is likely that the *N‐*benzyl or *N*‐phenylethyl substituents in compounds **21** are helping these compounds to bind to the viral proteins even if no substantial improvement from **6** was detected. A dose–response curve for hit compound **6** is reported in Figure 4.

**Figure 4:**
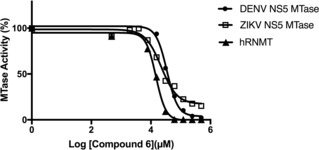
Dose‐response curve for hit compound **6**.

Finally, the ability of all the derivatives synthesized in this work to inhibit ZIKV replication was assessed. Results of the most active compounds, as well as their effect on cell viability, are reported in Table 2. Compound **6** and **9 a**–**d**, which showed MTase inhibitory activity, were devoid of any antiviral activity against ZIKV.

**Table 2:** EC_50_ and CC_50_ values against ZIKV of the most active compounds of the library.

Compd	ZIKV	
	EC_50_ [μM]	CC_50_ [μM]
6	>3.99	3.99
9a	>50	30.7
9b	>10	>10
9c	>10	>10
9d	>50	>50
11b	4.78	19.57
15	1.67	>12.5
21b	12.5	20
21d	25	100
21f	>20	20

Compd	DENV	
	EC_50_ [μM]	CC_50_ [μM]
15	9.75	20

Possible reasons for the lack of antiviral activity may be the inability of the molecules to enter the cell, or their conversion into inactive metabolites in infected cells. However, since compound **6** proved to be cytotoxic (CC_50_>3.99 μM), it is likely that this compound and analogues thereof can enter the cells. Moreover, the inhibitory activity of **6** and **9 a**–**d** against the host MTase (Table 1) hints toward an antimetabolic effect. Another possible explanation is that compounds **6** and **9 a**–**d** mainly inhibit the 2′‐O MTase activity without affecting the N7 MTase activity. This could explain the very poor inhibition of ZIKV replication in the cell‐based assay, as the 2′‐O methylation of the cap is not essential for viral replication in most cell lines. Derivative **11 b**, which showed some MTase inhibitory activity, also exhibited antiviral activity against ZIKV in the cell‐based assay (EC_50_=4.78 μM). Interestingly, derivative **15**,^31^ which did not show any activity against ZIKV NS5‐MTase, was able to inhibit ZIKV replication with an EC_50_ of 1.67 μM. It is plausible that **15** inhibits ZIKV replication via a different mode of action than via NS5‐MTase inhibition, or that this compound is partially metabolized in the treated cells.

Interestingly, compound **15** also showed some activity against DENV (EC_50_=9.75 μM), even if the antiviral activity is clearly linked to an adverse toxic effect on the host cell (CC_50_=20 μM). Finally, compound **21 b** which showed no enhanced activity against ZIKV MTases, proved to inhibit ZIKV replication at good concentration (EC_50_=12.5 μM). However, compound **21 b** showed toxicity in cellular assay as well as **21 f** which was found active on ZIKV MTase instead. This could suggest that these compounds might target another MTase (i. e. cellular MTase) involved in virus replication. Interestingly, compound **21 d**, which showed poor inhibition of ZIKV MTase, inhibits ZIKV with good EC_50_=25 μM and no toxicity (CC_50_=100 μM).

In summary, a structure‐based virtual screening protocol on a set of chemical libraries using the ZIKV NS5‐MTase was performed leading to the identification of a novel class of carbazoyl‐urea inhibitors. Compounds **6** and **21 c**, **21 e**, **21 f** showed inhibitory activity against ZIKV MTase as well as the related DENV MTase. Conversely, the urea derivative **15** showed an antiviral effect against ZIKV with EC_50_ of 1.67 μM, despite its poor inhibitory activity toward the NS5‐MTase *in vitro*. Studies to identify novel MTase inhibitors, to optimize the structure‐activity relationships of carbazoyl‐urea analogues of **6** and to fully unravel their mode of action are currently in progress in our laboratories.

## Experimental Section

Experimental details are reported in the Supporting Information.

## Conflict of interest

The authors declare no conflict of interest.

## Supporting information

As a service to our authors and readers, this journal provides supporting information supplied by the authors. Such materials are peer reviewed and may be re‐organized for online delivery, but are not copy‐edited or typeset. Technical support issues arising from supporting information (other than missing files) should be addressed to the authors.

Supplementary
