# Supplementary material for: Design, Synthesis and Discovery of N,N’‐Carbazoyl‐aryl‐urea Inhibitors of Zika NS5 Methyltransferase and Virus Replication
Source: ChemMedChem. 2020 Jan 7;15(4):385–90. doi: 10.1002/cmdc.201900533 (PMC7106487; doi:10.1002/cmdc.201900533)
Supplement: Supplementary file 1 — Supplementary [file CMDC-15-385-s001.pdf]

CHEMMEDCHEM

Supporting Information

Design, Synthesis and Discovery of *N,N′*-Carbazoyl-aryl-urea Inhibitors of Zika NS5 Methyltransferase and Virus Replication

Sharon Spizzichino, Giulio Mattedi, Kate Lauder, Coralie Valle, Wahiba Aouadi, Bruno Canard, Etienne Decroly, Suzanne J. F. Kaptein, Johan Neyts, Carl Graham, Zakary Sule, David J. Barlow, Romano Silvestri, and Daniele Castagnolo*

Materials and methods

Virtual screening and docking

Homology modelling and system preparation

The sequence of ZIKV NS5 methyltransferase was downloaded from GenBank (Accession code AY632535).[1] The structure of the protein was modeled with SWISS-MODEL, which identified PDB 4K6M (Japanese Encephalitis Virus NS5)[2] as best template, with an identity of 70%. Protein and ligands were then prepared with MOE 2014.[3] The protein residues were protonated with the Structure Preparation tool and partial charges were assigned using the AMBER99 forcefield.[4] Ligands were protonated accordingly, charges were assigned with the AM1-BCC method[5] and the conformations were energy minimized with the MMFF94x forcefield.[6]

Docking

The National Cancer Institute (NCI) Diversity Set V was docked in the NS5 model. Molegro Virtual Docker 5.5[7] was used for the virtual screening: the MolDockScore evaluator was employed with a grid resolution of 0.3 A, and 10 runs per compound were performed using the MolDock SE search algorithm, with a population size of 50, maximum iteration number of 1500 and energy threshold of 100. Post-docking hydrogen bond minimization was allowed. Internal hydrogen bonding and sp^2-sp^2 torsions were enabled. The search space was defined as a sphere with a radius of 15 A centered around the center of mass of the co-crystallized S-adenosyl methionine. 10 final poses per ligand were generated by clustering the conformations with a RMSD cutoff of 1 A.

For the docking of the urea derivatives, PLANTS 1.2[8] was used with a search space radius of 20A and a search speed of 1. Using the chemPLP scoring function, 10 poses per ligand were generated by clustering with a RMSD cutoff of 2 A.

Biology

DENV, ZIKV and hRNMT MTase production and purification

The coding sequence of the ZIKV MTase domain was cloned in pDest 14 (Invitrogen) expression vector as recently described,[9] and the DENV and ZIKV MTase were produced and purified following the protocols applied for the DENV MTase.[10] The human N7-MTase (hRNMT) was cloned in pDest 14 (Invitrogen) expression vector as described.[11] The hRNMT was produced in *e.coli* Rosetta (DE3) cells transformed with pDest14/6His-hRNMT and purified on immobilized-metal-affinity chromatography (IMAC, Amersham Biosciences) followed by hearine, as described previously.[11]

MTase inhibition assay

The MTase activity of the different recombinant proteins was assayed upon pre-incubation of the MTase (5 min) with a selected fragment and the reactions were started by addition of a

small capped RNA substrate $GpppAC_4$ with the $[^3H]$AdoMet.[12] The MTase activity assay was performed in 20 µl samples containing 40 mM Tris-HCl pH 7.5, 5 mM DTT (except for hRNMT), 10 µM AdoMet (0.2-2 µCi $[^3H]$SAM), 1 µM of DENV or ZIKV MTase or 200 nM of hRNMT, 1 µM $GpppAC_4$ in presence of 1µl of compounds solubilized in DMSO 100%. Reactions were incubated at 30°C for 30 min and stopped by 20-fold dilution in an ice-cold 100 µM AdoHcy (SAH) solution. Samples were then transferred onto a DEAE membrane (DEAE Filtermat; Wallac) by using a Filtermat Harvester (Packard Instruments) washed with 0.01 M ammonium formate (pH 8.0), water and 95 % ethanol, and the radioactivity transferred onto RNA was measured using a Wallac 1450 MicroBeta Trilux Liquid Scintillation Counter.

The inhibitor concentration at 50% activity (IC_{50}) was determined by performing the MTase assays in presence of serial dilution of the inhibitor in DMSO (final concentration in the assay 5%). All data points were measured in duplicate. The IC_{50} values were determined by curve fitting with Prism software using a logistic dose-response function: % activity = $100/(1+[I]/IC_{50})^b$, where b corresponds to the slope factor and [I] to inhibitor concentration.

CPE-reduction assay

The antiviral activity against ZIKV was assessed in a cell-based assay, essentially as described previously.[13]

Chemistry

Materials and methods

^{1}H-NMR and ^{13}C-NMR spectra were recorded ^{1}H Nuclear Magnetic Resonance (NMR) spectra were measured with an Ascend 400 spectrometer Bruker, Germany at room temperature (rt) operating at the frequencies indicated. Chemical shifts (δ) are in ppm, referenced to tetramethylsilane. Coupling constants (J) are reported in Hertz. Splitting patterns are abbreviated as follows: singlet (s), doublet (d), triplet (t), quartet (q), multiplet (m), sextet (sxt), broad (br) or some combination of them. Mass spectra (HRMS) were recorded at the EPSRC National Mass Spectrometry Service Centre on a Thermo Scientific LTQ Orbitrap XL Mass Spectrometer using low-resolution ESI or high-resolution nano ESI techniques. The purity of the compounds was assessed by reverse-phase liquid chromatography coupled with a mass spectrometer (Agilent series 1100 LC/MSD) with a UV detector at λ = 254 nm and an electrospray ionization source (ESI). HPLC analysis was carried out using a Perkin-Elmer 1100 HPLC system coupled with UV/Vis set to 254 nm. Mass spectra were acquired in positive mode scanning over the mass range of 50–1500. The following ion source parameters were used: drying gas flow, 9 mL/min; nebulize pressure, 40 psig; and drying gas temperature, 350 ºC. All target compounds possessed a purity of ≥ 95% as verified by HPLC analyses. TLC was performed using commercially available pre-coated plates and visualized with UV light at 254 nm; $KMnO_4$ was used to reveal the products. Flash column chromatography was carried out using Sigma Aldrich silica gel particle size, 40-63 µm particle size 60 Å. All reactions were conducted under a nitrogen atmosphere in oven-dried glassware unless stated otherwise. All solvents and commercially available reagents were used as received.

Synthesis of the carbazole derivative 1-(9-ethyl-9H-carbazol-3-yl)-3-(2-methyl-4-nitrophenyl)urea (6)

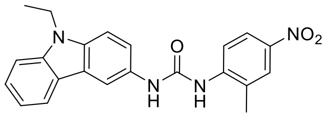

Under anhydrous conditions, 2-methyl-4 nitroaniline (200 mg, 0.95 mmol, 1 eq) was dissolved in THF and resulting solution was added dropwise into a stirred solution of triphosgene (281.91 mg, 0.95 mmol, 1eq) and anhydrous THF (5 mL). Afterward, triethylamine (201.7 mg, 2 mmol, 2.1 eq) was added, and the resulting mixture was left stirring for 2 h at 0 °C under a nitrogen atmosphere. Once the reaction had been finished as indicated by TLC, the mixture was concentrated *in vacuo*. The crude mixture, containing the isocyanate intermediate, was then dissolved in 5 mL of toluene and the 3-amino-9-ethyl carbazole (202 mg, 0.95 mmol, 1 eq) was slowly added into the flask. The reaction mixture was warmed to 60 ° C and left stirring under anhydrous conditions for 24 h. After the completion of the reaction, the mixture was concentrated *in vacuo* and the crude was purified by column chromatography using hexane/EtOAc 9:1 as eluent. Carbazole **6** was obtained with 73% yield.

Yield 73%. **^{1}H NMR** (400 MHz, DMSO-d_6) δ 9.45 (s, 1H), 8.50 (d, *J* = 9.08 Hz, 1H), 8.44 (s, 1H), 8.37 (s, 1H), 8.09 - 8.21 (m, 3H), 7.61 - 7.65 (m, 2H), 7.48 - 7.54 (m, 2H), 7.24 (t, *J* = 7.43 Hz, 1H), 4.48 (q, *J* = 7.03 Hz, 2H), 2.41 - 2.49 (m, 3H), 1.37 (t, *J* = 7.06 Hz, 3H) ppm. **^{13}C NMR** (101 MHz, DMSO-d_6) δ 152.3, 144.9, 140.6, 139.9, 135.9, 130.8, 126.3, 125.8, 125.4, 122.6, 122.1, 121.9, 120.3, 118.6, 118.4, 117.7, 110.7, 109.2, 109.1, 36.9, 17.8, 13.7 ppm. **HRMS** (m/z) $[M + H]^+$ calcd for $C_{22}H_{21}N_4O_3^+$, 389.1608; found, 389.1609.

Synthesis of the carbazole derivatives 9a-f and 11a-c

Under anhydrous conditions the appropriate isocyanate or isothiocyanate (200 mg, 0.8-1.2 mmol, 1 eq) and 3-amino-9-ethyl carbazole (168-252 mg, 0.8-1.2 mmol, 1 eq) were mixed in toluene (5 mL) and heated at 60 °C. The reaction mixture was stirred for 6h in a nitrogen atmosphere. Once the reaction was finished as indicated by TLC, the mixture was concentrated in vacuo and then purified by flash chromatography using hexane/EtOAc 9:1 as eluent.

1-(2-chloro-4-nitrophenyl)-3-(9-ethyl-9H-carbazol-3-yl)urea (9a)

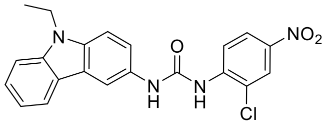

Yield 34%. **^{1}H NMR (**400 MHz, DMSO-d_6) δ 9.77 (s, 1H), 8.85 (br. s., 1H), 8.64 (d, *J* = 9.32 Hz, 1H), 8.34 (dd, *J* = 2.27, 9.32 Hz, 2H), 8.22 (dd, *J* = 2.64, 9.32 Hz, 1H), 8.12 (d, *J* = 7.68 Hz, 1H), 7.58 (d, *J* = 8.56 Hz, 2H), 7.41 - 7.49 (m, 2H), 7.18 (t, *J* = 7.43 Hz, 1H), 4.43 (q, *J* = 7.05 Hz, 2H), 1.31 (t, *J* = 7.05 Hz, 3H) ppm. **^{13}C NMR** (101 MHz, DMSO-d_6) δ 151.7, 142.8, 140.7, 140.0, 136.0, 130.4, 125.8, 124.8, 123.6, 122.1, 121.9, 120.4, 120.3, 118.7, 118.6, 118.5, 110.9, 109.3, 109.1, 36.9, 13.7 ppm. **HRMS** (m/z) $[M + H]^+$ calcd for $C_{21}H_{18}ClN_4O_3^+$, 409.1062; found, 409.1063.

Synthesis of 1-(4-cyanophenyl)-3-(9-ethyl-9H-carbazol-3-yl)urea (9b)

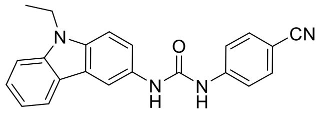

Yield 91%. ^{1}**H NMR** (400 MHz, DMSO-d6) δ 9.23 (s, 1 H), 8.83 (s, 1 H), 8.27 (d, *J* = 1.8 Hz, 1 H), 8.10 (d, *J* = 7.6 Hz, 1 H), 7.74 (d, *J* = 8.8 Hz, 2 H), 7.67 (d, *J* = 8.8 Hz, 2 H), 7.57 (t, *J* = 8.8 Hz, 2 H), 7.42 - 7.47 (m, 2 H), 7.17 (t, *J* = 7.3 Hz, 1 H), 4.42 (q, *J* = 6.9 Hz, 2 H), 1.30 (t, *J* = 7.1 Hz, 3 H), ppm. ^{13}**C NMR** (101 MHz, DMSO-d6) δ 152.5, 144.5, 140.6, 139.9, 135.9, 133.2, 130.7, 126.4, 125.7, 122.0, 121.9, 120.3, 119.4, 118.4, 117.9, 116.2, 109.1, 102.8, 36.4, 16.4 ppm. **HRMS** (m/z) $[M + H]^+$ calcd for $C_{22}H_{19}N_4O^+$, 355.1553; found, 355.1557.

1-(2,4-dimethoxyphenyl)-3-(9-ethyl-9H-carbazol-3-yl)urea (9c)

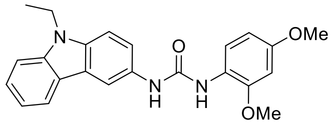

Yield 21%. ^{1}**H NMR** (400 MHz, CHLOROFORM-d) δ 7.96 - 8.24 (m, 2H), 7.85 - 7.96 (m, 2H), 7.32 - 7.56 (m, 4H), 7.21 (dd, *J* = 3.40, 8.94 Hz, 1H), 6.50 (dd, *J* = 2.58, 9.00 Hz, 1H), 6.45 (s, 1H), 4.37 (dd, *J* = 7.43, 14.86 Hz, 2H), 3.79 (s, 3H), 3.71 (s, 3H), 1.44 (t, *J* = 7.18 Hz, 4H). ^{13}**C NMR** (101 MHz, DMSO-d6) δ 155.1, 153.5, 149.5, 140.4, 135.9, 132.4, 126.1, 126.0, 122.7, 122.6, 122.5, 120.7, 120.0, 119.7, 118.8, 110.5, 109.6, 109.5, 104.6, 55.7, 55.3, 37.4, 14.1 ppm. **HRMS** (m/z) $[M + H]^+$ calcd for $C_{23}H_{24}N_3O_3^+$, 390.1812; found, 390.1814.

1-(9-ethyl-9H-carbazol-3-yl)-3-(4-iodophenyl)urea (9d)

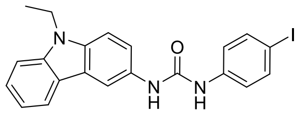

Yield 12%. ^{1}H NMR (400 MHz, DMSO-d_6) δ 8.87 (s, 1H), 8.72 (s, 1H), 8.26 (d, *J* = 1.89 Hz, 1H), 8.09 (d, *J* = 7.55 Hz, 1H), 7.58 - 7.62 (m, 3H), 7.52 - 7.58 (m, 2H), 7.43 - 7.46 (m, 1H), 7.33 - 7.38 (m, 2H), 7.17 (s, 1H), 4.42 (q, *J* = 7.05 Hz, 2H), 0.86 (t, *J* = 6.80 Hz, 3H). ^{13}**C NMR** (101MHz, DMSO-d6) δ 153.5, 140.8, 140.6, 140.4, 137.7, 136.2, 131.9, 126.1, 123.5, 123.2, 122.6, 122.5, 120.7, 120.6, 119.1, 118.8, 111.0, 109.6, 90.7, 37.4, 14.2 ppm. **HRMS** (m/z) $[M + H]^+$ calcd for $C_{21}H_{19}IN_3O^+$, 456.0567; found, 456.0565.

1-(9-ethyl-9H-carbazol-3-yl)-3-(4-(methylthio)phenyl)urea (9e)

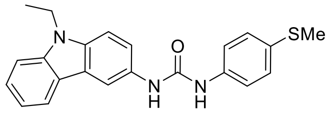

Yield 32 %. ^{1}**H NMR** (400 MHz, DMSO-d_6) δ 8.82 (s, 1H), 8.74 (s, 1H), 8.26 (d, *J* = 2.01 Hz, 1H), 8.09 (d, *J* = 7.55 Hz, 1H), 7.39 - 7.62 (m, 6H), 7.12 - 7.27 (m, 3H), 4.42 (q, *J* = 7.01 Hz, 2H), 2.45 (s, 3H), 1.30 (t, *J* = 7.11 Hz, 3H) ppm. ^{13}**C NMR** (101 MHz, DMSO-d_6) δ 153.0, 140.8, 140.6, 139.9, 137.8, 135.6, 135.1, 131.5, 129.4, 127.8, 125.6, 122.0, 122.0, 120.3, 120.2, 118.7, 118.3, 110.6, 109.0, 36.9, 16.0, 13.7 ppm. **HRMS** (m/z) $[M + H]^+$ calcd for $C_{22}H_{22}N_3OS^+$, 376.1478; found, 376.1476.

1-(9-ethyl-9H-carbazol-3-yl)-3-(4-(trifluoromethyl)phenyl)urea (9f)

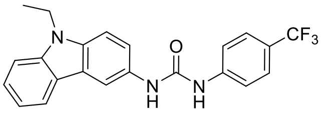

Yield 53%. **^{1}H NMR** (400 MHz, DMSO-d$_6$) δ 9.35 (s, 1H), 8.97 (s, 1H), 8.29 (d, *J* = 1.89 Hz, 1H), 8.10 (d, *J* = 7.55 Hz, 1H), 7.68 - 7.75 (m, *J* = 8.69 Hz, 2H), 7.62 - 7.68 (m, *J* = 8.81 Hz, 2H), 7.53 - 7.60 (m, 2H), 7.42 - 7.49 (m, 2H), 7.14 - 7.20 (m, 1H), 4.43 (q, *J* = 7.13 Hz, 2H), 1.31 (t, *J* = 7.11 Hz, 3H) ppm. **^{13}C NMR** (101 MHz, DMSO-d$_6$) δ 152.8, 143.9, 139.9, 135.8, 134.2, 131.1, 126.1, 126.0, 125.7, 123.3, 122.1, 122.0, 121.5, 120.3, 118.8, 118.4, 117.6, 110.9, 109.1, 36.9, 13.7 ppm. **HRMS** (m/z) [M + H]$^+$ calcd for $C_{22}H_{19}F_3N_3O^+$, 398.1475; found, 398.1475.

1-(9-Ethyl-9H-carbazol-3-yl)-3-phenylthiourea (11a)

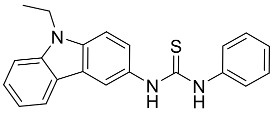

Yield 74%. **^{1}H NMR** (400 MHz, DMSO-d$_6$) δ 9.80 (s, 1H), 9.64 (s, 1H), 8.05 - 8.20 (m, 2H), 7.55 - 7.65 (m, 2H), 7.40 - 7.54 (m, 4H), 7.28 - 7.38 (m, 2H), 7.07 - 7.24 (m, 2H), 4.45 (q, *J* = 7.05 Hz, 2H), 1.32 (t, *J* = 7.18 Hz, 3H) ppm. **^{13}C NMR** (101 MHz, CDCl$_3$) δ 181.9, 140.4, 140.0, 128.8, 126.3, 124.7, 124.3, 120.9, 119.1, 117.8, 109.6, 109.3, 37.5, 14.2 ppm. **HRMS** (m/z) [M + H]$^+$ calcd for $C_{21}H_{20}N_3S^+$, 346.1372; found, 346.1375.

1-Benzyl-3-(9-ethyl-9H-carbazol-3-yl)thiourea (11b)

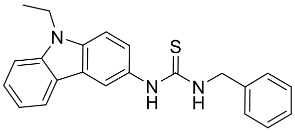

Yield 80%. **^{1}H NMR** (400 MHz, CHLOROFORM-d) δ 7.97 (d, *J* = 7.68 Hz, 1H), 7.88 (d, *J* = 2.01 Hz, 1H), 7.72 (s, 1H), 7.41 - 7.48 (m, 2H), 7.30 - 7.38 (m, 3H), 7.20 - 7.27 (m, 6H), 4.83 (d, *J* = 5.54 Hz, 2H), 4.30 (q, *J* = 7.30 Hz, 2H), 1.36 (t, *J* = 7.24 Hz, 3H) ppm. **^{13}C NMR** (101 MHz, CHLOROFORM-d) δ 181.9, 140.5, 139.0, 139.0, 137.5, 137.5, 128.7, 127.6, 127.5, 126.7, 126.4, 124.3, 123.8, 122.1, 120.6, 119.5, 118.9, 109.7, 108.9, 49.4, 37.7, 13.8 ppm. **HRMS** (m/z) [M + H]$^+$ calcd for $C_{22}H_{22}N_3S^+$, 360.1529; found, 360.1531.

Synthesis of *N,N'*-diaryl urea and thiourea derivatives 12a-e.

The appropriate substituted aniline (1 mmol, 1 eq) was dissolved in acetone (5 mL) under inert atmosphere (N_2) and added with an appropriate isocyanate or isothiocyanate (1 mmol, 1 eq). The mixture was stirred at room temperature and monitored via TLC. When completed, the reaction mixture was acidified with 1M HCl till pH 1-2 and then concentrated *in vacuo.* The mixture was then extracted with EtOAc (2x5 mL) and the combined organic layers were washed with brine (10 mL). The organic layers were dried over $MgSO_4$, filtered and evaporated under vacuum. The crude products were purified by flash chromatography using hexane/EtOAc 9:1 as eluent.

_N_-Benzyl-_N′_-(2,5-dimethylphenyl)thiourea (12a).

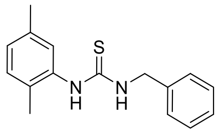

Yields 55 %. **^{1}H NMR** (400 MHz, DMSO-d6) δ 9.19 (br s, 1 H), 7.83 (br s, 1 H), 7.36 - 7.27 (m, 4 H), 7.27 - 7.22 (m, 1 H), 7.13 (d, *J* = 7.8 Hz, 1H) , 7.03 (d, *J* = 0.8 Hz, 1H), 6.99 (br d, *J* = 7.8 Hz, 1H) , 4.70 (br d, *J* = 3.8 Hz, 2H) , 2.25 (s, 3 H), 2.11 (s, 3 H) ppm. **^{13}C NMR** (101 MHz, DMSO-d_6) δ 181.9, 139.9, 136.2, 135.9, 132.1, 130.8, 128.8, 128.6, 127.8, 127.7, 127.2, 123.5, 123.2, 47.8, 20.9, 17.7 ppm. **HRMS** (m/z) $[M + H]^+$ calcd for $C_{16}H_{19}N_2S^+$, 271.1263; found, 271.1265.

_N_-Benzyl-_N′_-(4-chlorophenyl)thiourea (12b).[14]

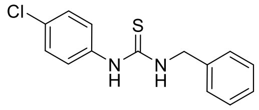

^{1}H NMR (400 MHz, DMSO-d6) δ 9.70 (br s, 1H), 8.28 (br s, 1H), 7.48 (d, *J* = 8.6 Hz, 2H), 7.37 (d, *J* = 9.1 Hz, 2H), 7.35 (m, *J* = 2.3 Hz, 3H), 7.23 - 7.30 (m, 2H), 4.73 (d, 2H, *J* = 4.5 Hz) ppm. **LRMS** (ESI+): m/z = 277 $[M + H]^+$.

_N_-(4-fluorophenyl)-_N′_-phenylthiourea (12c).[15]

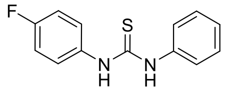

^{1}H NMR (400 MHz, DMSO-d6) δ 9.78 (s, 1H), 9.73 (s, 1H), 7.49-7.46 (m, 4H), 7.36-7.32 (m, 2H),7.19-7.12 (m, 3H) ppm. **LRMS** (ESI+): m/z = 247 $[M + H]^+$.

_N_-(2-chlorophenyl)-_N′_-naphthalen-1-ylurea (12d).

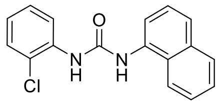

Yields 35 %. **^{1}H NMR** (400MHz, DMSO-d6) δ 9.45 (bs, 1H), 8.81 (s, 1H), 8.24 - 8.17 (m, 2H), 8.01 (dd, *J* = 0.9, 7.7 Hz, 1H), 7.98 - 7.92 (m, 1H), 7.67 (d, *J* = 8.1 Hz, 1H), 7.64 - 7.59 (m, 1H), 7.56 (s, 1H), 7.53 - 7.46 (m, 2H), 7.32 (s, 1H), 7.08 - 7.01 (m, 1H) ppm. **^{13}C NMR** (101MHz, DMSO-d6) δ 152.6, 141.7, 136.0, 133.9, 133.6, 129.2, 128.3, 127.5, 126.0, 125.9, 125.8, 125.7, 123.3, 123.2, 121.9, 121.5, 118.0 ppm. **HRMS** (m/z) $[M + H]^+$ calcd for $C_{17}H_{14}ClN_2O^+$, 297.0789; found, 297.0787.

_N_-(4-chlorophenyl)-_N′_-(4-cyanophenyl)urea (12e).

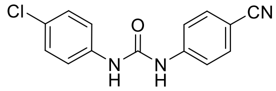

Yields 43 %. **^{1}H NMR** (400 MHz, DMSO-d6) 9.24 (s, 1H), 9.01 (s, 1H), 7.73 (d, 2H, *J* = 8.8 Hz), 7.64 (d, 2H, *J* = 9.3 Hz), 7.50 (d, 2H, *J* = 8.8 Hz), 7.35 (d, 2H, *J* = 9.1 Hz) ppm. **^{13}C NMR** (101 MHz, DMSO-d6) δ 151.9, 144.0, 138.1, 133.3, 128.7, 125.9, 120.0, 119.3, 118.1, 103.3 ppm. **HRMS** (m/z) $[M + H]^+$ calcd for $C_{14}H_{11}ClN_3O^+$, 272.0585; found, 272.0588.

Synthesis of 5-isocyanato-1-methyl-1*H*-indole derivatives (14a-d)

Under anhydrous conditions, the 5-isocyanato-1-methyl-1H-indole (100 mg, 0.58 mmol, 1 eq) was dissolved in toluene (10 mL) and the appropriate aniline (0.58 mmol, 1 eq) was added dropwise. The resulting reaction mixture was heated to 60 °C and allowed to stir for 8 hours. Once the reaction was completed, as indicated by TLC, the mixture was concentrated *in vacuo* and the product was purified with flash column chromatography using EtOAc/Methanol 9:1 as eluent.

1-(1-Methyl-1H-indol-5-yl)-3-phenylurea (14a).

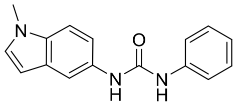

Yield 64%. **^{1}H NMR** (400 MHz, DMSO-d6) δ 8.57 (s, 1H), 8.43 (s, 1H), 7.69 (d, *J* =1.76 Hz, 1H), 7.37 - 7.21 (m, 4H) 7.51 - 7.40 (m, 2H), 7.14 (dd, *J* = 8.69, 2.01 Hz, 1H), 6.99 - 6.90 (m, 1H), 6.35 (dd, *J* = 3.02, 0.76 Hz, 1H), 3.76 (s, 3H) ppm. **^{13}C NMR** (101 MHz, DMSO-*d*$_6$) δ 153.4, 140.6, 139.37, 133.3, 132.1, 130.5, 129.2, 128.5, 121.9, 118.9, 118.4, 115.1, 110.6, 110.0, 100.4, 32.9 ppm. **HRMS** (m/z) $[M + H]^+$ calcd for $C_{16}H_{16}N_3O^+$, 266.1288; found, 266.1291.

1-(4-Fluorophenyl)-3-(1-methyl-1H-indol-5-yl)urea (14b).

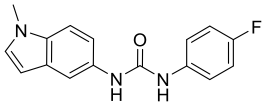

Yield 76%. **^{1}H NMR** (400 MHz, DMSO-d6) δ 8.63 (s, 1H), 8.45 (s, 1H), 7.68 (d, *J* = 1.76 Hz, 1H), 7.54 - 7.41 (m, 2H), 7.34 (d, *J* = 8.81 Hz, 1H), 7.27 (d, *J* = 3.02 Hz, 1H), 7.18 - 7.06 (m, 3H), 6.34 (dd, *J* = 2.96, 0.57 Hz, 1H), 3.76 (s, 3H) ppm. **^{13}C NMR** (101 MHz, DMSO-d6) δ 153.0, 136.5, 136.4, 132.8, 131.5, 129.9, 119.6, 119.6, 115.3, 115.1, 114.6, 110.1, 109.5, 99.9, 32.5 ppm. **HRMS** (m/z) $[M + H]^+$ calcd for $C_{16}H_{15}FN_3O^+$, 284.1194; found, 284.1196.

1-(2,4-Dichlorophenyl)-3-(1-methyl-1H-indol-5-yl)urea (14c).

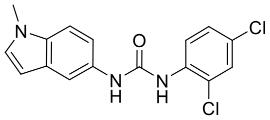

Yield 24%. **^{1}H NMR** (400 MHz, DMSO-*d*$_6$) δ 9.35 (s, 1H), 8.36 (s, 1H), 8.25 (d, *J* = 9.06 Hz, 1H), 7.75-7.67 (m, 1H), 7.61 (d, *J* = 2.39 Hz, 1H), 7.43 - 7.22 (m, 3H), 7.20 - 7.08 (m, 1H), 6.40 - 6.27 (m, 1H), 3.77 - 3.75 (m, 3H) ppm. **^{13}C NMR** (101 MHz, DMSO-*d*$_6$) δ 152.8, 136.1, 133.5, 131.6, 130.6, 128.9, 128.6, 128.1, 126.1, 122.7, 122.3, 114.9, 110.7, 110.2, 100.5, 32.9 ppm. **HRMS** (m/z) $[M + H]^+$ calcd for $C_{16}H_{14}ClN_3O^+$, 334.0508; found, 334.0505.

1-(2,5-Dimethylphenyl)-3-(1-methyl-1H-indol-5-yl)urea (14d).

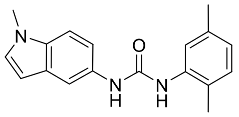

Yield 79%. **^{1}H NMR** (400 MHz, DMSO-d6) δ 9.02 (s, 1H), 7.91 (s, 1H), 7.81 - 7.71 (m, 2H), 7.34 (d, *J* = 8.69 Hz, 1H), 7.26 (d, *J* = 2.90 Hz, 1H), 7.16 (dd, *J* = 8.69, 1.76 Hz, 1 H), 7.03 (d,

J = 7.55 Hz, 1H), 6.73 (d, *J* = 7.43 Hz, 1H), 6.34 (d, *J* = 2.77 Hz, 1 H), 3.76 (s, 3H), 2.25 (s, 3H), 2.21 (s, 3H) ppm. **^{13}C NMR** (101 MHz, DMSO-d_6) δ 153.5, 138.1, 135.4, 133.2, 132.4, 130.4, 130.4, 128.6, 124.3, 123.3, 121.7, 114.8, 110.2, 110.0, 100.4, 32.9, 21.5, 18.0 ppm. **HRMS** (m/z) $[M + H]^+$ calcd for $C_{18}H_{20}N_3O^+$, 294.1601; found, 294.1605.

Synthesis of 1-(4-amino-2-methylphenyl)-3-(9-ethyl-9H-carbazol-3-yl)urea (15)

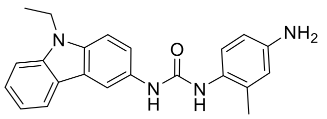

Compound **6** (50 mg, 0.13 mmol, 1 eq) was dissolved into a mixture of DCM (2 mL), concentrated HCl (12N, 0.30 ml, 85.6 eq) and MeOH (1 mL). Iron fillings were then added (49.38 mg, 0.88 mmol, 49 eq) and the mixture was left stirring at 40 °C for 24 h. Once the reaction was completed as indicated by TLC, the mixture was poured into cold H_2O and basified to pH>8 with NaOH. The mixture was then extracted with DCM (2x5 mL). The combined organic phases were dried over anhydrous $MgSO_4$, filtered and concentrated in vacuo. The crude compound was purified with flash column chromatography using EtOAc/Methanol 9:1 as eluent.

Yield 45%. **^{1}H NMR** (400 MHz, DMSO-d_6) δ 8.57 (s, 1H), 8.37 - 8.48 (m, 1H), 8.23 (s, 1H), 8.03 - 8.16 (m, 2H), 7.56 - 7.61 (m, 1H), 7.53 (d, *J* = 9.90 Hz, 1H), 7.41 - 7.51 (m, 3H), 7.15 - 7.22 (m, 2H), 6.33 - 6.45 (m, 2H), 4.73 - 4.83 (m, 2H), 4.37 - 4.47 (m, 2H), 2.41 (s, 1H), 1.31 (q, *J* = 6.94 Hz, 3H) ppm. **^{13}C NMR** (101 MHz, DMSO-d_6) δ 152.8, 149.7, 145.3, 139.2, 136.4, 130.6, 126.3, 125.9, 123.1, 122.6, 120.6, 119.0, 118.3, 116.0, 113.9, 112.2, 111.3, 109.8, 102.6, 37.4, 18.5, 14.2 ppm. **HRMS** (m/z) $[M + H]^+$ calcd for $C_{22}H_{23}N_4O^+$, 359.1866; found, 359.1869.

Synthesis of 2,6-diamino-N-(9-ethyl-9H-carbazol-3-yl)hexanamide (16)

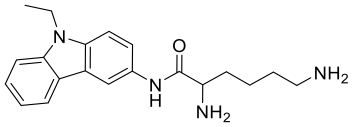

9-ethyl-9H-carbazol-3-amine (54.67 mg, 0.26 mmol, 1 eq) and 6-(((benzyloxy)carbonyl)amino)-2-((tert butoxycarbonyl)amino)-hexanoic acid (100 mg, 0.26 mmol, 1eq) were dissolved in a solution containing 1-ethyl-3-(3-dimethylaminopropyl)-carbodiimide (EDC, 59.8 mg, 0.31mmol, 1.20 eq), hydroxybenzotrazole (HOBt, 45.67 mg, 0.33 mmol, 1.30 eq), N,N-diisopropylethylamine (DIPEA, 54 ul, 0.31 mmol, 1.20 eq) and DMF (3 mL). The reaction mixture was introduced into a microwave vessel and irradiated for 10 minutes at 60 °C (250 psi). The crude mixture was then extracted with EtOAc (2x5 mL). The combined organic layers were washed with brine (10 mL), dried over anhydrous MgSO4, filtered and concentrated in vacuo. The crude product was then dissolved in HCl/EtOAc solution (2 mL) in a sealed flask and the reaction was stirred at room temperature for two days. The solvent was then evaporated and the compound 16 was obtained as pure HCl salt.

Yield 62%. **^{1}H NMR** (400 MHz, CD_3OD) δ 8.45 (d, *J* = 15.77 Hz, 1H), 8.06 (d, *J* = 6.97 Hz, 1H), 7.60 - 7.70 (m, 1H), 7.46 - 7.53 (m, 2H), 7.36 (s, 1H), 7.25 (s, 1H), 7.21 (s, 1H), 4.39 - 4.49 (m, 2H), 3.20 (s, 1H), 3.01 (m, 2H), 1.87 (m, 2H), 1.53 - 1.69 (m, 4H), 1.42 (m, 3H) ppm. **^{13}C NMR** (101 MHz, CD_3OD) δ 166.7, 137.4, 128.0, 127.5, 125.8, 122.7, 119.9, 119.2, 118.6, 112.4, 112.2, 108.6, 108.5, 53.6, 39.2, 37.1, 31.1, 26.9, 21.8, 12.7 ppm. **HRMS** (m/z) $[M + H]^+$ calcd for $C_{20}H_{27}N_4O^+$, 339.2179; found, 339.2181.

Synthesis of 3-nitro-9H-carbazole (18)[16]

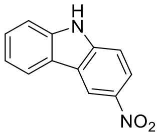

A mixture of 9H-carbazole (4 g, 23.9 mmol, 1 eq), concentrated nitric acid (60% HNO3, 4ml, 86 mmol, 3.6 eq) and distilled water (30 mL) was stirred for 1 h at room temperature. Then, the temperature of the reaction was raised at the rate of 10 °C per hour and kept at 80 °C for 3 h. After completion, the reaction mixture was cooled and poured into 100 mL of water. The solid product was then filtered off and washed with water. The compound was dried at 60 °C for 24 h in the oven. The dried powder was purified by flash chromatography using EtOAc/MeOH 9:1 as eluent to afford 18 with 32% yield.
^{1}H NMR (400 MHz, DMSO-d_6) δ 12.08 (s, 1H), 9.17 (d, *J* = 2.27 Hz, 1H), 8.38 (d, *J* = 7.81 Hz, 1H), 8.30 (dd, *J* = 2.33, 9.00 Hz, 1H), 7.58 - 7.67 (m, 2H), 7.52 (dt, *J* = 1.13, 7.62 Hz, 1H), 7.26 - 7.33 (m, 1H) ppm. **LRMS** (ESI+): m/z = 213 $[M + H]^+$.

Synthesis of carbazoles 19a-b

Under anhydrous conditions, carbazole **18** (500 mg, 2.36 mmol, 1eq) and sodium hydride (60 % dispersion in mineral oil; 115.2 mg, 2.83 mmol, 1.2 eq) were dissolved in dry DMF (5 mL). The mixture was stirred for 30 minutes at room temperature. Benzylchloride or (2-bromoethyl)benzene (406- 792 ml, 3.53- 5.56 mmol, 1.5 eq) and KI (10mol%) were slowly added to the reaction mixture which was then heated to 80 °C and left stirring for 2 h under a nitrogen atmosphere. The reaction was quenched with water (10 mL) followed by addition of ethyl acetate (10 mL). The aqueous layer was extracted with ethyl acetate (2x5mL) and the organic layers were then washed with deionized water (2x10 mL) and brine (15 mL). The organic phase was dried over anhydrous $MgSO_4$, filtered, concentrated in vacuo and purified by flash column chromatography using hexane/EtOAc 2:8 as eluent.

9-Benzyl-3-nitro-9H-carbazole (19a)

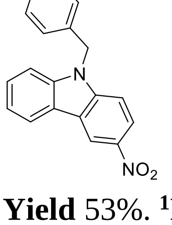

Yield 53%. **^{1}H NMR** (400 MHz, $CDCl_3$) δ 9.08 (d, *J* = 2.02 Hz, 1H), 8.38 (dd, *J* = 2.15, 9.03 Hz, 1H), 8.22 (d, *J* = 7.70 Hz, 1H), 7.52 - 7.60 (m, 1H), 7.47 (d, *J* = 8.25 Hz, 1H), 7.37 - 7.44 (m, 2H), 7.27 - 7.34 (m, 5H), 5.60 (s, 2H) ppm. **^{13}C NMR** (101 MHz, DMSO-d_6) δ 141.9,

140.9, 138.7, 134.0, 129.0, 128.9, 127.6, 127.2, 127.1, 125.7, 123.4, 122.4, 120.5, 118.5, 115.8, 110.3, 109.6, 104.8, 46.0 ppm. **LRMS** (ESI+): m/z = 303 $[M + H]^+$.

3-Nitro-9-phenethyl-9H-carbazole (19b)

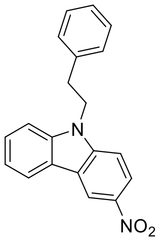

Yield 53%. **^{1}H NMR** (400 MHz, $CDCl_3$) δ 8.91 (d, *J* = 2.02 Hz, 1H), 8.19 (dd, *J* = 8.99, 1.93 Hz, 1H), 8.19 (dd, *J* = 8.99, 1.93 Hz, 1H), 8.07 (d, *J* = 7.79 Hz, 1H), 7.42 -7.51 (m, 1H), 7.30 - 7.36 (m, 1H), 7.27 (t, *J* =7 .47 Hz, 1H), 7.09 - 7.16 (m, 2H), 7.07 (d, *J* = 8.99 Hz, 1H), 6.94-7.03 (m, 2H), 4.49 (t, *J* = 7.15 Hz, 2H), 3.08 (t, *J* = 7.15 Hz, 2H) ppm. **^{13}C NMR** (100 MHz, $CDCl_3$) δ 143.4, 141.3, 140.6, 137.9, 128.7, 128.7, 127.3, 127.0, 122.8, 122.4, 121.5, 121.0, 120.8, 117.2, 109.5, 108.1, 45.4, 35.1 ppm. **LRMS** (ESI+): m/z = 317 $[M + H]^+$.

Synthesis of carbazol-3-amines 20a-b.

The carbazoles **19a-b** (200 mg, 2.12 mmol, 1 eq) were dissolved in a HCl 12N (2 mL)/ glacial AcOH (8 mL) solution and $SnCl_2$ (923 mg, 4.92 mmol, 5 eq) was added. The mixture was stirred at room temperature for 30 minutes, then the temperature was raised to 100 °C for 12 h. The reaction mixture was then basified (pH=14) with NaOH 2N, extracted with ethyl acetate (2x5 mL). The organic layers were washed with deionized water (10 mL), dried over anhydrous $MgSO_4$, filtered and concentrated in vacuo. The product was used in the next step without any further purification.

9-Benzyl-9H-carbazol-3-amine (20a)

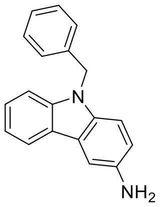

^{1}H NMR (400 MHz, DMSO-d_6) δ 7.97 (d, *J* = 7.79 Hz, 1H), 7.50 (d, *J* = 8.25 Hz, 1H), 7.09 - 7.32 (m, 9H), 6.83 (dd, *J* = 1.74, 8.53 Hz, 1H), 5.53 (s, 2H), 4.97 (br. s., 2H) ppm. **LRMS** (ESI+): m/z = 273 $[M + H]^+$.

9-Phenethyl-9H-carbazol-3-amine 20b

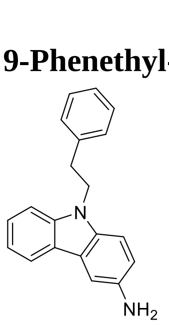

^{1}H NMR (400 MHz, DMSO-d_6) d 7.97 (d, *J* = 7.79 Hz, 1H), 7.50 (d, *J* = 8.25 Hz, 1H), 7.09 - 7.32 (m, 9H), 6.83 (dd, *J* = 1.74, 8.53 Hz, 1H), 5.53 (s, 2H), 4.97 (br. s., 2H), 1.05 - 1.32 (m, 2H) ppm. **LRMS** (ESI+): m/z = 287 $[M + H]^+$.

Synthesis of ureas 21a-b

Under anhydrous conditions, 2 methyl-4 nitroaniline (37 mg, 0.24 mmol, 1.38 eq.) was dissolved in THF and resulting solution was slowly dropped into a stirred solution of triphosgene (73.10 mg, 0.25 mmol, 1.38 eq.) and THF (5 ml). Afterward, Et_3N (2.9 eq) was added to the reaction mixture, which was left stirring for 4 h at 0°C under a nitrogen atmosphere. Once the reaction was finished as indicated by TLC, the mixture was concentrated in vacuo. The crude product (isocyanate intermediate) was dissolved in 5 mL of toluene and **20a-b** (0.18 mmol, 1 eq) was slowly added to the flask. The reaction mixture was heated at 60 °C and left stirring under anhydrous conditions for 12 h. The mixture was then concentrated in vacuo and the crude product was purified by column chromatography using EtOAc/Methanol 9:1 as eluent.

1-(9-Benzyl-9H-carbazol-3-yl)-3-(2-methyl-4-nitrophenyl)urea (21a)

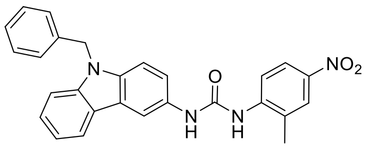

^{1}H NMR (400 MHz, DMSO-d_6) δ 9.49 (s, 1H), 8.45 (s, 1H), 8.43 (s, 1H), 8.34 - 8.38 (m, 1H), 8.09 - 8.17 (m, 3H), 7.55 - 7.64 (m, 2H), 7.39 - 7.47 (m, 2H), 7.25 - 7.31 (m, 2H), 7.20 - 7.24 (m, 2H), 7.18 (d, *J* = 7.79 Hz, 2H), 5.65 (s, 2H), 2.41 (s, 3H) ppm. **^{13}C NMR** (100 MHz, $CDCl_3$) δ 153.0, 148.0, 145.5, 141.0, 138.3, 136.9, 131.9, 129.0, 127.6, 127.2, 127.0, 126.3, 125.9, 122.9, 122.7, 120.7, 119.3, 119.0, 118.3, 110.9, 110.1, 110.0, 46.1, 18.8 ppm. **HRMS** (m/z) $[M + H]^+$ calcd for $C_{27}H_{23}N_4O_3^+$, 451.1765; found, 451.1769

1-(2-Methyl-4-nitrophenyl)-3-(9-phenethyl-9H-carbazol-3-yl)urea (21b).

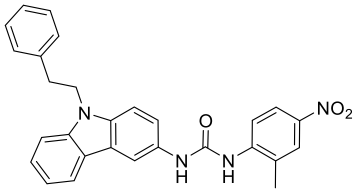

Yield 23%.**^{1}H NMR** (400 MHz, DMSO-d_6) δ 9.49 (s, 1H), 8.41 - 8.46 (m, 2H), 8.31 (d, *J* = 1.65 Hz, 1H), 8.08 - 8.14 (m, 3H), 7.53 (dd, *J* = 2.57, 8.44 Hz, 3H), 7.43 (dd, *J* = 1.97, 8.85 Hz, 2H), 7.24 - 7.27 (m, 3H), 7.16 - 7.20 (m, 2H), 4.59 (t, *J* = 7.34 Hz, 2H), 3.03 - 3.08 (m, 2H), 2.41 (s, 3H) ppm. **^{13}C NMR** (101 MHz, DMSO-d_6) δ 152.7, 141.2, 140.8, 139.1, 136.7, 129.4, 128.8, 126.8, 126.2, 125.9, 123.1, 122.6, 122.4, 120.7, 119.0, 118.3, 116.6, 111.1, 110.0, 109.8, 46.3, 33.4, 18.3 ppm. **HRMS** (m/z) $[M + H]^+$ calcd for $C_{28}H_{25}N_4O_3^+$, 465.1921; found, 465.1925.

Synthesis of ureas 21c-f.

The appropriate isocyanate (179-245 mg, 0.18 mmol, 1 eq) and **20a-b** (272-286 mg, 0.18 mmol, 1 eq) were mixed in toluene (10 mL) and the resulting solution was heated at 60 °C for

12 h under a nitrogen atmosphere. Once the reaction had ended, the mixture was concentrated in vacuo and the crude product was purified by flash chromatography using hexane/EtOAc 9:1 as eluent.

1-(9-Benzyl-9H-carbazol-3-yl)-3-(4-iodophenyl)urea (21c)

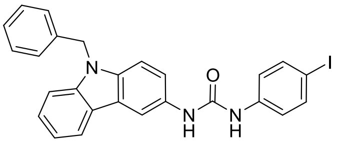

Yield 53%. **^{1}H NMR** (400 MHz, DMSO-d$_6$) δ 8.89 (br. s., 1H), 8.74 (br. s., 1H), 8.29 (d, *J* = 1.65 Hz, 1H), 8.12 (d, *J* = 7.70 Hz, 1H), 7.57 - 7.63 (m, 3H), 7.55 (d, *J* = 8.80 Hz, 1H), 7.38 - 7.45 (m, 2H), 7.35 (d, *J* = 8.80 Hz, 2H), 7.24 - 7.30 (m, 2H), 7.20 - 7.24 (m, 1H), 7.17 (t, *J* = 6.88 Hz, 3H), 5.63 (s, 2H) ppm. **^{13}C NMR** (101 MHz, DMSO-d$_6$) δ 153.4, 141.1, 140.5, 139.6, 138.4, 137.8, 136.8, 132.1, 129.0, 127.7, 127.7, 127.2, 126.3, 122.7, 122.6, 120.8, 120.8, 120.7, 119.5, 119.3, 111.4, 111.3, 111.3, 110.1, 84.7, 46.1 ppm. **HRMS** (m/z) $[M + H]^+$ calcd for $C_{26}H_{21}IN_3O^+$, 518.0724; found, 518.0731.

1-(4-Iodophenyl)-3-(9-phenethyl-9H-carbazol-3-yl)urea (21d).

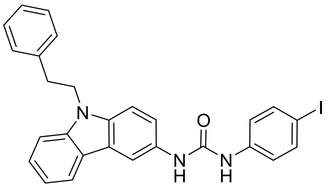

Yield 62 %. **^{1}H NMR** (400 MHz, DMSO-d$_6$) δ 8.16 (s, 1H), 7.99 (s, 1H), 7.38 (s, 1H), 7.21 (d, *J* = 7.70 Hz, 1H), 6.75 (d, *J* = 8.44 Hz, 2H), 6.61 - 6.68 (m, 2H), 6.48 - 6.56 (m, 4H), 6.36 - 6.43 (m, 4H), 6.27 - 6.34 (m, 2H), 3.71 (t, *J* = 7.06 Hz, 2H), 2.18 (t, *J* = 6.51 Hz, 2H) ppm. **^{13}C NMR** (101 MHz, DMSO-d$_6$) δ 153.4, 140.7, 140.5, 137.8, 131.9, 129.4, 128.8, 126.8, 126.1, 122.5, 122.4, 120.8, 120.6, 119.1, 118.9, 111.0, 109.8, 84.5, 55.4, 34.9 ppm. **HRMS** (m/z) $[M + H]^+$ calcd for $C_{27}H_{23}IN_3O^+$, 532.0880; found, 532.0888.

1-(9-Benzyl-9H-carbazol-3-yl)-3-(2,4-dimethoxyphenyl)urea (21e)

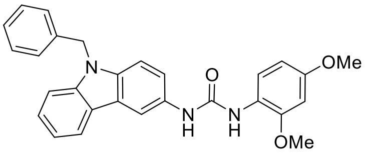

Yield 32%. **^{1}H NMR** (400 MHz, DMSO-d$_6$) δ 9.13 (s, 1H), 8.31 (s, 1H), 8.11 (d, *J* = 7.70 Hz, 1H), 7.97 (br. s., 1H), 7.52 - 7.64 (m, 2H), 7.35 - 7.45 (m, 2H), 7.14 - 7.30 (m, 7H), 6.63 (d, *J* = 2.20 Hz, 1H), 6.50 (dd, *J* = 2.48, 8.71 Hz, 1H), 5.63 (s, 2H), 3.88 (s, 3H), 3.75 (s, 3H) ppm. **^{13}C NMR** (101 MHz, DMSO-d$_6$) δ 162.6, 153.6, 150.9, 150.3, 149.5, 141.1, 138.4, 129.0, 127.7, 127.2, 126.3, 122.7, 120.7, 120.1, 119.2, 116.1, 110.0, 104.6, 99.3, 56.3, 55.8, 46.1 ppm. **HRMS** (m/z) $[M + H]^+$ calcd for $C_{28}H_{26}N_3O_3^+$, 452.1969; found, 452.1971.

1-(2,4-Dimethoxyphenyl)-3-(9-phenethyl-9H-carbazol-3-yl)urea (21f)

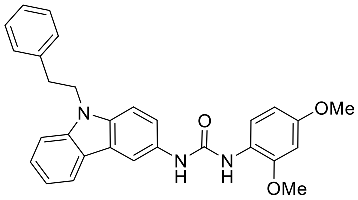

Yield 52%. **^{1}H NMR** (400 MHz, DMSO-d_6) δ 8.25 (s, 1H), 7.40 (d, *J* = 1.74 Hz, 1H), 7.20 (d, *J* = 7.70 Hz, 1H), 7.08 - 7.15 (m, 2H), 6.62 (d, *J* = 8.89 Hz, 1H), 6.66 (d, *J* = 8.34 Hz, 1H), 6.49 - 6.57 (m, 2H), 6.36 - 6.43 (m, 3H), 6.27 - 6.35 (m, 2H), 5.78 (d, *J* = 2.57 Hz, 1H), 5.64 (dd, *J* = 2.48, 8.89 Hz, 2H), 3.71 (t, *J* = 7.29 Hz, 2H), 3.03 (s, 3H), 2.89 (s, 3H), 2.19 (t, *J* = 7.20 Hz, 2H) ppm. **^{13}C NMR** (101 MHz, DMSO-d_6) δ 171.4, 155.2, 149.5, 139.2, 136.2, 132.9, 132.5, 132.4, 129.4, 128.8, 128.7, 126.8, 126.2, 126.1, 123.0, 122.5, 122.4, 120.6, 120.1, 118.9, 118.7, 110.4, 109.8, 104.6, 99.3, 56.3, 55.4, 44.6, 34.9 ppm. **HRMS** (m/z) $[M + H]^+$ calcd for $C_{29}H_{28}N_3O_3^+$, 466.2125; found, 466.2129.

References

1) www.ncbi.nlm.nih.gov/genbank
2) G. Lu, P. Gong, *PLoS Pathog.* **2013**, 9, e1003549.
3) Molecular Operating Environment (MOE), 2013.08; Chemical Computing Group ULC, 1010 Sherbooke St. West, Suite #910, Montreal, QC, Canada, H3A 2R7, 2018.
4) D. A. Case, T. E. Cheatham, T. Darden, H. Gohlke, R. Luo, K. M. Merz, A. Onufriev, C. Simmerling, B. Wang, and R. J. Woods, *J. Comput. Chem.* **2005**, *26*, 1668.
5) A. Jakalian, B. L. Bush, D. B. Jack, C. I. Bayly, *J. Comput. Chem.* **2000**, *21*, 132.
6) T. A. Halgren, *J. Comput. Chem.* **1996**, *17*, 490.
7) R. Thomsen, M. H. Christensen, *J. Med. Chem.* **2006**, *49*, 3315.
8) O. Korb, T. Stützle, T. E. Exner, *Lecture Notes in Computer Science* **2006**, *4150*, 247-258.
9) B. Coutard, K. Barral, J. Lichière, B. Selisko, B. Martin, W. Aouadi, M. O. Lombardia, F. Debart, J. J. Vasseur, J. C. Guillemot, B. Canard, E. Decroly, *J. Virol.* **2017**, *91*, e02202-16.
10) K. Barral, C. Sallamand, C. Petzold, B. Coutard, A. Collet, Y. Thillier, J. Zimmermann, J. J. Vasseur, B. Canard, J. Rohayem, F. Debart, E. Decroly, *Antiviral Res.* **2013**, *99*, 292-300.
11) F. Peyrane, B. Selisko, E. Decroly, J. J. Vasseur, D. Benarroch, B. Canard, K. Alvarez *Nucleic Acids Res.* **2007**, *35*, e26.
12) B. Selisko, F. F. Peyrane, B. Canard, K. Alvarez, E. Decroly, *J. Gen. Virol.* **2009**, *91*, 112-121.
13) J. Zmurko, R. E. Marques, D. Schols, E. Verbeken, S. J. F. Kaptein, J. Neyts, *PLoS Negl. Trop. Dis.* (May 10) **2016**.
14) R. Raja, D. Murugan, A, Sivasubramaniyan, J, George, S. Perumal, P. Alagusundaram, M. R. Jayakumar, M. Saminathan, *Synth. Commun.* **2016**, *46*, 942-948.
15) V. Štrukil, M. D. Igrc, L. Fábián, M. Eckert-Maksić, S. L. Childs, D. G. Reid, M. J. Duer, I. Halasz, C. Mottilloe, T. Friščić, *Green Chem.* **2012**, *14*, 2462-2473.
16) S.-K. Ku, J.-H. Lee, Y. O, W. Lee, G.-Y. Song, J.-S. Bae, *Bioorg. Med. Chem. Lett.* **2015**, *25*, 4304-4307.
